# Glycemic and Lipid Outcomes Associated with Bitter Melon Blood-Sugar-Modulating Protein and Metformin Use: A Translational Preclinical and Retrospective Study

**DOI:** 10.3390/nu18162649

**Published:** 2026-08-13

**Authors:** Pei-Yung Liao, Hsin-Ting Yu, Tse-Yen Yang, Hsin-Yi Lo, Chien-Yun Hsiang, Tin-Yun Ho

**Affiliations:** 1 Division of Endocrinology and Metabolism, Department of Internal Medicine, Changhua Christian Hospital, Changhua 500209, Taiwan; 48795@cch.org.tw; 2 Department of Nutrition and Dietetics of Healthcare System, Changhua Christian Hospital, Changhua 500209, Taiwan; 43070@cch.org.tw; 3 Center for General Education, Taipei Medical University, Taipei 110301, Taiwan; hardawayoung@gmail.com; 4 Graduate Institute of Chinese Medicine, China Medical University, Taichung 404328, Taiwan; hsinyilo0123@gmail.com; 5 Department of Microbiology and Immunology, China Medical University, Taichung 404328, Taiwan; 6 Office of Research & Development, Asia University, Taichung 413305, Taiwan

**Keywords:** *Momordica charantia*, blood-sugar modulating protein, metformin, glycemic control

## Abstract

**Background/Objectives:** Interactions between botanical supplements and standard antidiabetic therapies remain poorly understood. This study investigated the efficacy and mechanistic properties of a bitter melon blood-sugar-modulating protein (BMP) in relation to concurrent metformin use for improving glucose and lipid metabolism. **Methods:** A translational approach combined mechanistic evaluation in *db*/*db* mice with an exploratory, real-world observational cohort study of 119 individuals with prediabetes or diabetes. BMP was characterized by liquid chromatography-electrospray ionization-tandem mass spectrometry. In the retrospective self-controlled clinical arm, participants receiving daily BMP supplementation were stratified by metformin use and dosage to evaluate changes in glycated hemoglobin (HbA1c) and lipid profiles over 3 months. **Results:** In *db*/*db* mice, BMP significantly reduced fasting blood glucose and HbA1c, with greater glucose-lowering efficacy observed when combined with metformin compared to metformin alone (approximately 300 mg/dL vs. 200 mg/dL reduction; *p* < 0.05). Skeletal muscle transcriptomic analysis identified coordinated regulation of 27 shared pathways involved in glucose and lipid metabolism. In the clinical cohort, BMP supplementation was associated with reductions in HbA1c (−0.23%, *p* = 0.0028), total cholesterol (−7.5 mg/dL), and low-density lipoprotein cholesterol (−6.1 mg/dL) among metformin users, whereas no significant changes were observed in non-users. The greatest observed HbA1c reduction (−0.32%, *p* < 0.0001) occurred in participants receiving 1000 mg/day of metformin. **Conclusions:** Preclinical data demonstrate coordinated pathway remodeling in skeletal muscle and improved glycemic control when BMP is combined with metformin. In the clinical cohort, BMP supplementation was associated with modest reductions in HbA1c and lipid parameters, primarily among participants using concurrent metformin. Given the retrospective, self-controlled design and baseline clinical differences between treatment groups, these clinical findings reflect observational associations rather than causal effects. Overall, this study is the first to demonstrate that BMP, a major protein component of bitter melon, exhibits complementary metabolic effects when combined with first-line antidiabetic therapy in animal models. Furthermore, by revealing a corresponding observational association in human clinical settings, this work establishes an important foundation for nutritional protein research.

## 1. Introduction

Diabetes mellitus is a major metabolic disorder characterized by chronic hyperglycemia and progressive metabolic dysfunction [1]. According to the International Diabetes Federation, approximately 589 million adults were living with diabetes in 2024, with over 3.4 million deaths directly attributed to diabetes, highlighting it as a global health threat [2]. Metformin remains the first-line pharmacological therapy for type 2 diabetes mellitus (T2DM), primarily acting through suppression of hepatic gluconeogenesis and improvement of insulin sensitivity [3]. However, progressive β-cell dysfunction often necessitates combination therapy to achieve adequate glycemic control, highlighting the need for complementary therapeutic strategies [4].

Increasing attention has been directed toward dietary and nutraceutical interventions as adjuncts for glycemic management [5,6,7,8,9,10]. Evidence suggests that specific dietary components and supplements, including mango, dragon fruit, barley fiber, kale, raspberry leaf tea polyphenols, chromium, vitamin D and magnesium, can improve glucose homeostasis and insulin sensitivity [11,12,13,14,15,16,17]. Nevertheless, most studies have evaluated these interventions in isolation, with limited investigation into their interactions with standard antidiabetic medications. Given that patients commonly use nutraceuticals alongside pharmacotherapy, the potential for pharmacodynamic interactions remains insufficiently characterized.

*Momordica charantia* (bitter melon) is widely consumed as both a functional food and traditional antidiabetic remedy, with clinical studies supporting its glucose-lowering effects [18,19,20,21,22,23]. However, clinical evidence remains mixed, with several trials reporting modest, inconsistent, or non-significant changes in glycated hemoglobin (HbA1c) and fasting glucose [20,22,23,24]. A primary driver of this inconsistency is product heterogeneity; most commercial and clinical evaluations utilize unstandardized whole-fruit powders or crude aqueous/alcoholic extracts with poorly characterized bioactive profiles and variable batch-to-batch consistency [24]. Furthermore, safety concerns and potential supplement–drug interactions warrant critical attention. Although generally well tolerated, bitter melon administration has been associated with gastrointestinal adverse events (e.g., diarrhea, abdominal pain), rare reports of hepatotoxicity, and favism in glucose-6-phosphate dehydrogenase-deficient individuals [24]. Crucially, co-administration of bitter melon with standard oral hypoglycemic agents (such as metformin or sulfonylureas) presents an unpredictable risk of additive hypoglycemia or altered drug metabolism via modulation of intestinal transporters and cytochrome P450 enzymes [24]. To address these limitations, isolating and characterizing specific bioactive molecules is essential. In experimental in vitro and animal models, previous studies have identified an abundant bitter melon blood-sugar-modulating protein (BMP) and its derived gastro-resistant peptides that lower glucose through a proposed “second-hit” mechanism, defined preclinically as binding to a secondary, distinct site on the insulin receptor (IR) to directly potentiate IR autophosphorylation and downstream substrate signaling, independently of metformin’s AMPK-mediated pathway [25,26,27,28,29]. While these cell and rodent studies demonstrate a distinct mode of action, whether this experimentally observed receptor activation translates into complementary metabolic benefits in clinical settings remains unknown.

To bridge this gap, the present study establishes a novel translational framework that connects preclinical skeletal muscle transcriptomic profiling with real-world clinical observations. Unlike previous trials investigating crude extracts or our group’s prior structural and bioactivity evaluations of BMP in isolation, this work specifically evaluates BMP as a pharmaconutritional adjunct alongside background metformin therapy. By contrasting BMP monotherapy with combination regimens across both molecular pathway networks and observational clinical outcomes, this study provides a distinct perspective on the complementary application of peptide-based nutraceuticals in diabetes management. Specifically, we developed a standardized BMP-enriched preparation and characterized the BMP via liquid chromatography-electrospray ionization-tandem mass spectrometry (LC-ESI-MS/MS) to evaluate its metabolic effects alongside metformin therapy. We hypothesized that the IR-potentiating effects observed preclinically for BMP would complement metformin-mediated pathways, leading to favorable metabolic changes when used concurrently. To test this, we employed a translational strategy integrating mechanistic evaluation in a T2DM mouse model with real-world clinical evidence from a self-controlled retrospective cohort. By evaluating skeletal muscle signaling preclinically and analyzing longitudinal glycemic and lipid changes across background metformin regimens clinically, this study aims to evaluate shared metabolic pathways and explore observational associations between BMP supplementation and clinical parameters in a real-world setting.

## 2. Materials and Methods

### 2.1. Preparation and Standardization of BMP

*Momordica charantia* Linn. fruits were sourced from a Good Agricultural Practices-certified farm (Taichung, Taiwan) and harvested 12 weeks post-planting. The fruits were dried at 60 °C and subjected to aqueous extraction as previously described [26,30]. The resulting extract was lyophilized to produce BMP powder. Absorbance scanning of the BMP powder demonstrated a primary peak at 280 nm, confirming peptide/protein dominance. Approximately 4 mg BMP was encapsulated in semi-transparent capsules under HACCP- and ISO-compliant manufacturing conditions. To ensure quality and reproducibility across experimental applications, quality control and batch release standards were applied. Total protein purity of approximately 99% was routinely verified using sodium dodecyl sulfate-polyacrylamide gel electrophoresis (SDS-PAGE) coupled with densitometric analysis and confirmed by LC-ESI-MS/MS peptide mapping. Batch consistency across independent production lots was strictly maintained, achieving an acceptable coefficient of variation of <5.0% for total protein content. Stability testing demonstrated that the lyophilized BMP powder remains stable, maintaining >95% intact protein integrity and retaining IR-activation potency when stored at room temperature for up to 36 months. Furthermore, each production batch underwent comprehensive food microbiological testing and pesticide residue analysis by SGS (Taichung, Taiwan) to confirm compliance with human dietary supplement standards prior to cohort administration.

### 2.2. Characterization of BMP

BMP powder was suspended in distilled water and centrifuged at 12,000× *g* for 15 min at 4 °C to remove insoluble material. The supernatant was collected for subsequent analyses. Protein composition was initially evaluated by spectrophotometry (Multiskan Go, Thermo Fisher Scientific, Waltham, MA, USA) over a wavelength range of 240–600 nm. Protein profiles were further analyzed by SDS-PAGE using 15% resolving gels. Gels were stained with Coomassie Brilliant Blue, and band intensities were quantified using Gel-Pro Analyzer software (version 3.0; Media Cybernetics, Rockville, MD, USA).

To simulate gastrointestinal digestion, BMP was subjected to sequential enzymatic digestion using pepsin and pancreatin, as previously described [29]. Briefly, samples were adjusted to pH 2.0 and incubated with pepsin at 37 °C for 30 min. The pH was then adjusted to 7.5, followed by incubation with pancreatin at 37 °C for 60 min. The reaction was terminated by adding 100 mM phenylmethylsulfonyl fluoride to a final concentration of 1 mM. The resulting peptides were analyzed by LC-ESI-MS/MS using a quadrupole time-of-flight mass spectrometer (Applied Biosystems, Foster City, CA, USA). MS/MS spectra were searched against the BMP sequence (GenBank accession no. AGU04661.1) using the MASCOT search engine (version 2.5, Matrix Science, Boston, MA, USA). Search parameters included no enzyme specificity (unspecific digestion), a precursor mass tolerance of 10 ppm, and a fragment mass tolerance of 0.02 Da. Peptide identifications were filtered at a false discovery rate (FDR) of <1% and only high-confidence matches were retained for further analysis. Peptide abundance was estimated based on spectral counts. The base peak chromatogram and total ion chromatogram, used to assess LC-MS/MS performance and signal stability, are provided in Supplementary Figure S1.

### 2.3. Animal Experiment

Diabetic *db*/*db* mice (BKS.Cg-*Dock7*^m^ +/+ *Lepr*^db^/JNarl, 8-week-old, female, 35–40 g) were purchased from the National Center for Biomodels (Taipei, Taiwan). Female mice were used to minimize variability associated with aggressive behavior and metabolic differences. Mice were maintained under a 12:12 light-dark cycle with free access to water and food unless otherwise indicated. All experimental procedures were approved by the Institutional Animal Care and Use Committee of China Medical University (Permit No. CMUIACUC-2022-284-1) and conducted in accordance with the U.S. National Institutes of Health Guidelines for the Care and Use of Laboratory Animals (NIH Publication No. 85–23, revised 1996).

Mice were excluded prior to treatment if baseline fasting blood glucose was <250 mg/dL. To minimize bias, mice were allocated into four groups using a computer-generated block randomization sequence (n = 7 per group): distilled water (mock group), 10 mg/kg metformin (metformin group), 10 mg/kg BMP (BMP group), and 10 mg/kg BMP plus 10 mg/kg metformin (BMP + metformin group). Treatments were administered by oral gavage once daily for 8 consecutive weeks. Dose selection for BMP and metformin was based on previous studies demonstrating metabolic efficacy [27,31]. Body weight and food intake were measured every week. Blood samples were collected every four weeks from the tail vein after an overnight fast (12–16 h). Fasting blood glucose levels were measured using an IME-DC blood glucose monitor (Arctic Medical Ltd., Folkestone, UK). HbA1c levels were measured by immunoassay using a DCA Vantage Analyzer (Siemens, Munich, Germany). Outcome assessors conducting fasting blood glucose tests, HbA1c assays, and transcriptomic data processing were blinded to group allocations throughout testing and analysis. No animal mortality or adverse events occurred during the experiment; zero attrition was recorded, and all biological samples (n = 7 per group) were included in all final analyses.

### 2.4. RNA-Seq Analysis

After an 8-week treatment, mice were euthanized under 4% isoflurane anesthesia (Baxter, Deerfield, IL, USA). Total RNAs were extracted from skeletal muscle tissue (n = 3 biological replicates per group) using a RNeasy Mini kit (Qiagen, Hilden, Germany). RNA concentration and integrity were assessed using a spectrophotometer and the Agilent 2100 Bioanalyzer (Agilent Technologies, Santa Clara, CA, USA). Only samples meeting an RNA Integrity Number threshold of ≥7 were selected for library preparation. Standard poly(A) mRNA selection library preparation was performed, and cDNA libraries were sequenced on an Illumina platform (Illumina, San Diego, CA, USA) with 150 bp paired-end reads at an average sequencing depth of >20 million reads per sample, as previously described [32]. All samples were extracted, prepared, and sequenced in a single batch to avoid batch-related variation.

Read quality pre- and post-filtering was verified using FastQC (v0.11.9). High-quality reads were aligned to the mouse reference genome build (GRCm38/mm10) using HISAT2 (v2.2.1). Gene expression levels were quantified using featureCounts (Subread package v2.0.3) against GenCode mouse annotation (vM25). Genes with low abundance (<10 raw counts across all samples) were filtered prior to statistical analysis. Transcripts Per Million were calculated to normalize for gene length and sequencing depth. Differential expression was evaluated using the EBSeq package (v1.38.0). Differentially expressed genes (DEGs) were identified based on FDR-adjusted *p* < 0.05 and absolute fold change ≥ 2 or ≤ −2. To determine true combination-specific transcriptional effects, direct contrast analyses were conducted comparing the combination treatment group against each individual monotherapy treatment group in addition to untreated controls. Kyoto Encyclopedia of Genes and Genomes (KEGG) pathway analyses were conducted via DAVID 2021 update (https://davidbioinformatics.nih.gov/; accessed on 1 May 2022) using Benjamini–Hochberg adjusted *p* < 0.05 [33]. Heatmaps were subsequently generated using Morpheus (https://software.broadinstitute.org/morpheus; accessed on 15 July 2022). The raw RNA-sequencing datasets generated and analyzed during the current study have been deposited in the NCBI Gene Expression Omnibus and are accessible under accession number (GSE341666).

### 2.5. Self-Controlled Retrospective Cohort Study

This study employed a self-controlled longitudinal design, in which each participant served as their own control across pre- and post-supplementation periods. Clinical records were generated between April 2021 and April 2022, during which patients routinely received BMP as part of standard clinical care. Formal ethical approval to retrospectively extract, de-identify, and analyze these records was granted by the Changhua Christian Hospital Institutional Review Board (IRB) on 17 February 2022 (CCH IRB No. 220132) in accordance with the Declaration of Helsinki. Patient consent was explicitly waived by the IRB due to the retrospective nature of the study and the use of de-identified electronic health record data.

### 2.6. Study Participants

This study retrospectively evaluated electronic health record data from 134 participants diagnosed with prediabetes or diabetes who used BMP capsules continuously for three months between April 2021 and April 2022 in the Department of Endocrinology and Metabolism at Changhua Christian Hospital (Changhua, Taiwan). BMP supplementation was prescribed/utilized independently as part of routine real-world clinical care, not as a prospective research intervention. All participants were aged between 20 and 80 years old. Participants who did not attend follow-up visits at least twice within one year were excluded. Of the initial 134 participants identified from routine clinical records, 119 met all inclusion criteria and were included in the final analysis.

### 2.7. Clinical Management and Outcome Measurement

Basic participant demographics and clinical characteristics, including ethnicity (100% Han Chinese population), age, gender, height, weight, body mass index (BMI), fasting blood glucose, diabetes type and diabetes duration, was recorded at baseline (0 month). Participants were instructed to consume one BMP capsule daily before breakfast for 3 months. For safety reasons, participants maintained their physician-prescribed antidiabetic regimens without alteration throughout the study. General lifestyle advice regarding diet and physical activity was provided according to standard outpatient clinical care guidelines, though formal quantitative tracking of daily caloric intake, exercise, or longitudinal weight changes was not systematically performed.

Clinical and biochemical parameters were evaluated at three time points: 3 months prior to supplementation (−3 months, pre-supplementation), baseline (0 month, initiation of BMP supplementation), and 3 months after supplementation (+3 months, post-supplementation). The period between −3 months and 0 month served as the 3-month pre-supplementation retrospective control period to establish intra-individual baseline trajectory prior to BMP initiation. In accordance with the IRB-approved data extraction protocol, clinical and biochemical parameters were retrieved retrospectively from electronic medical records. The primary outcome was HbA1c level. Secondary outcomes included lipid profile parameters, specifically total cholesterol, high-density lipoprotein cholesterol (HDL-C), and low-density lipoprotein cholesterol (LDL-C), as well as metformin dosage. Each participant served as their own internal control, with paired comparisons evaluated between pre-supplementation (−3 months) and baseline (0 month), as well as between baseline (0 month) and post-supplementation (+3 months).

### 2.8. Statistical Analysis and Sample Size Justification

Continuous variables are expressed as mean ± standard deviation (SD), median (interquartile range), or mean difference (Δ) with 95% confidence intervals (95% CI) based on normality testing via the Shapiro–Wilk test. Categorical variables are presented as counts and percentages.

For the preclinical study, n = 7 mice per group provided >80% power (α = 0.05) to detect a 20% difference in fasting blood glucose (assuming a 15% SD). Between-group comparisons among experimental groups (mock, metformin, and combination treatment) at each indicated time point were evaluated using one-way analysis of variance (ANOVA) with post-hoc Bonferroni corrections for normally distributed data or Kruskal–Wallis tests with Dunn’s post-hoc corrections for non-normally distributed data.

For the clinical cohort (N = 119), post-hoc power calculations indicated >85% power (α = 0.05) to detect primary longitudinal HbA1c changes, and n = 105 complete cases provided >80% power for lipid profiles. The clinical metformin threshold (1000 mg/day) was prespecified based on standard maintenance dosing guidelines. Missing data were confirmed to be missing completely at random due to routine clinical attrition rather than adverse events; thus, a complete-case analysis approach (n = 105 for lipids; n = 119 for primary glycemic endpoints) was applied without post-hoc imputation.

Longitudinal changes in continuous clinical parameters and lipid profile outcomes across key evaluation time points, between pre-supplementation (−3 months) and baseline (0 month), as well as baseline (0 month) and post-supplementation (+3 month), were evaluated using paired *t*-tests or Wilcoxon signed-rank tests for non-normally distributed continuous variables. Bonferroni corrections were applied to adjust for multiple comparisons across outcome parameters and sequential time points. Between-group differences across exposure categories were evaluated using independent *t*-tests or one-way ANOVA with post-hoc Bonferroni pairwise comparisons where applicable. Categorical variables were compared using Pearson’s chi-square test or Fisher’s exact test. Cohen’s *d* was calculated to evaluate clinical effect sizes.

To address cohort heterogeneity (N = 119, which included individuals with prediabetes and n = 2 with type 1 diabetes mellitus (T1DM)), a pre-planned sensitivity analysis was performed restricted strictly to patients with confirmed T2DM (n = 88). Multiplicity across secondary outcomes was further controlled using Benjamini–Hochberg FDR corrections. Statistical significance was set at a two-tailed *p* < 0.05 (or adjusted *p*-values following Bonferroni/FDR correction). All clinical data analyses were performed using IBM SPSS Statistics (version 28.0; IBM Corp., Armonk, NY, USA) and GraphPad Prism (version 9.0; GraphPad Software, San Diego, CA, USA).

## 3. Results

### 3.1. Characterization of BMP and Identification of Digestion-Derived Bioactive Peptides

**Figure 1:**
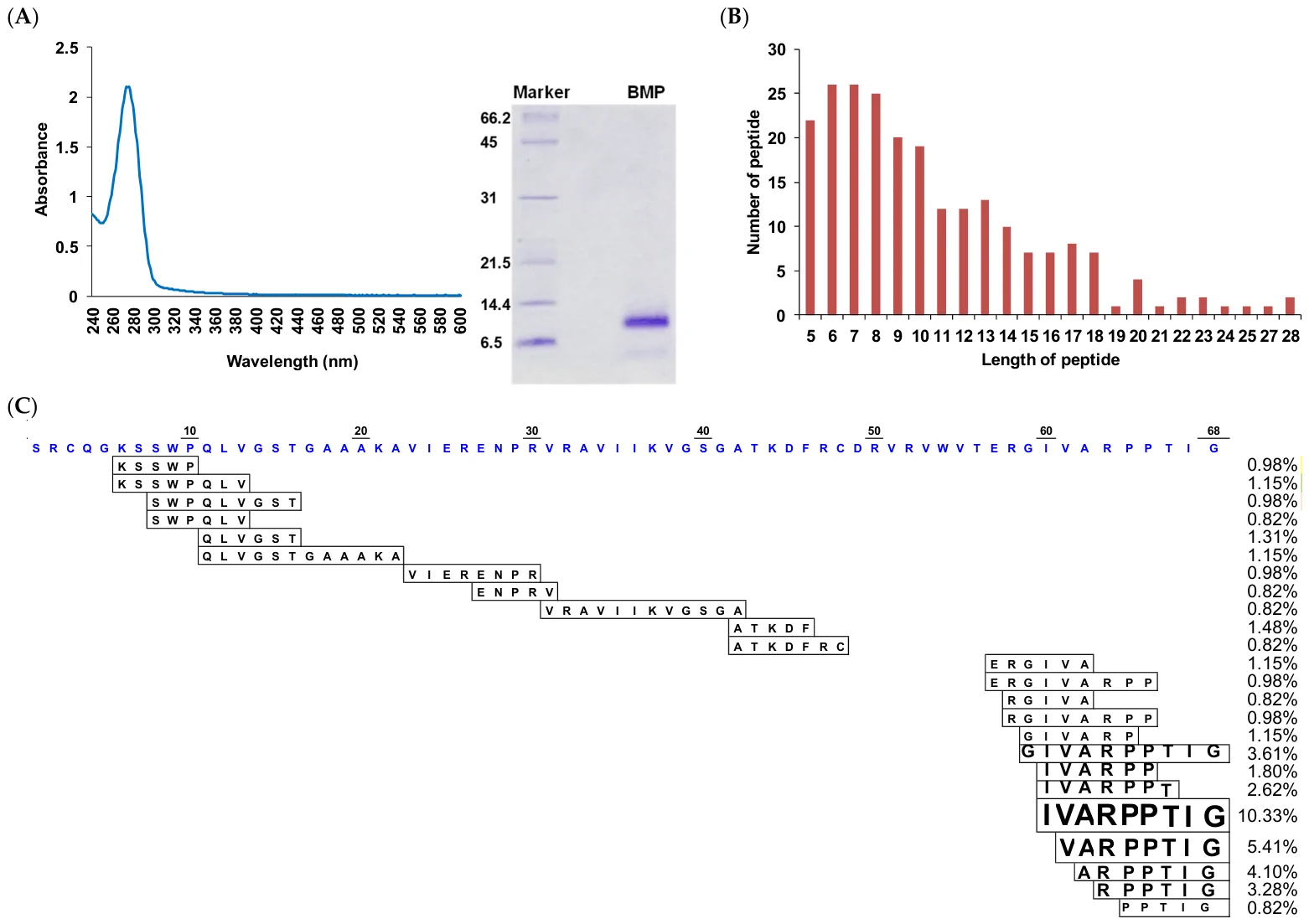
Characterization of BMP. (**A**) Spectrophotometric scanning (**left panel**) and SDS-PAGE analysis (**right panel**) of BMP. Proteins were visualized using Coomassie Brilliant Blue R-250. Protein size markers (in kDa) are indicated on the left. (**B**) Length distribution of peptides identified from BMP after in vitro digestion. The histogram displays the number of peptides identified by LC-ESI-MS/MS categorized by their amino acid length. (**C**) Mapping of major peptide fragments released from BMP after in vitro digestion. The primary sequence of BMP is shown at the top, with identified peptide fragments aligned to their relative positions. Only fragments with a spectral count >5 are displayed. The font size reflects the relative abundance. The percentages on the right indicate the frequency of each specific fragment relative to the total identified peptides.

In this study, we prepared a standardized BMP-enriched preparation and characterized BMP via spectrophotometric scanning, SDS-PAGE, and LC-ESI-MS/MS. The chemical components in BMP were first evaluated using spectrophotometric scanning and SDS-PAGE. A continuous wavelength scan from 240 to 600 nm revealed a maximal absorbance at 280 nm (Figure 1A), suggesting that the protein was the predominant component of the preparation. Consistently, 15% SDS-PAGE analysis showed a single dominant band at approximately 8 kDa, corresponding to the reported molecular weight of BMP. Densitometric analysis further demonstrated that ~99% of total protein content was concentrated in this band, confirming that bitter melon preparation was enriched with BMP.

To further identify the presence of BMP and characterize bioactive peptides released upon digestion, BMP was subjected to simulated gastrointestinal digestion using pepsin and pancreatin, followed by LC-ESI-MS/MS analysis. The base peak chromatogram revealed multiple peptide signals across the elution profile, indicating successful generation and separation of digestion-derived peptides (Supplementary Figure S1). Peptide length distribution analysis showed that the distribution was heavily skewed toward shorter fragments, ranging primarily from 5 to 10 amino acids (Figure 1B). The frequency of identified peptides decreased progressively with increasing sequence length, confirming that the protein underwent extensive proteolytic cleavage during the in vitro digestion process.

A total of 14,580 MS/MS spectra were acquired, of which 610 peptides were confidently matched to BMP sequences, yielding 229 unique peptides after redundancy removal. Peptide abundance, estimated by spectral counting, revealed a subset of dominant fragments (spectral count >5) (Figure 1C). Notably, the 9-amino-acid peptide IVARPPTIG was the most abundant, accounting for 10.33% of all identified sequences. IVARPPTIG has been previously reported as a gastro-resistant peptide derived from BMP with hypoglycemic activity mediated through interaction with the IR [29]. The high abundance of this peptide following in vitro digestion suggested that it represented a major bioactive component of BMP. Collectively, these results demonstrate that bitter melon preparation was enriched with BMP and that gastrointestinal digestion preferentially releases stable, low-molecular-weight peptides, among which IVARPPTIG is a potential candidate contributing to its metabolic activity.

### 3.2. BMP Enhanced Glycemic Control and Expanded Skeletal Muscle Transcriptional Responses When Combined with Metformin

**Figure 2:**
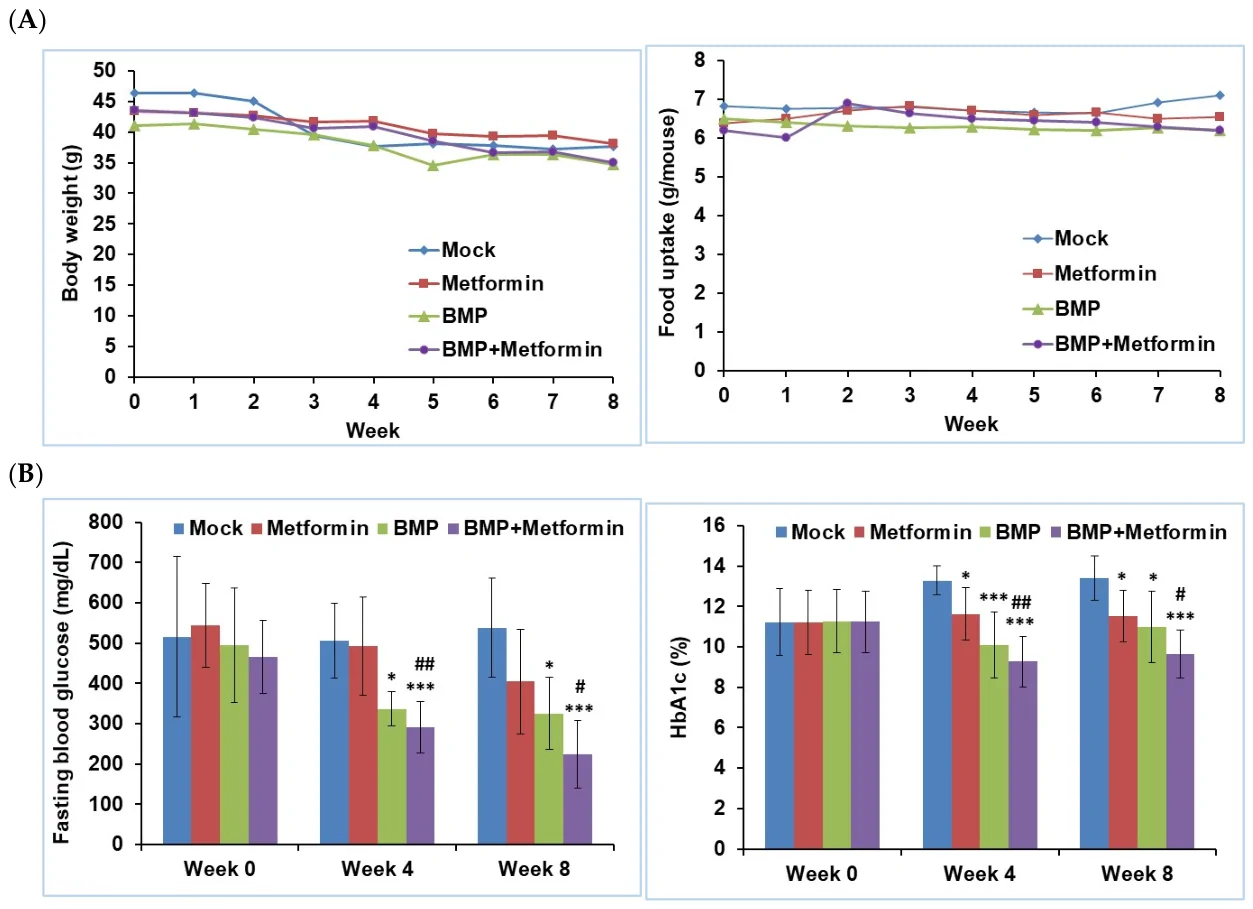

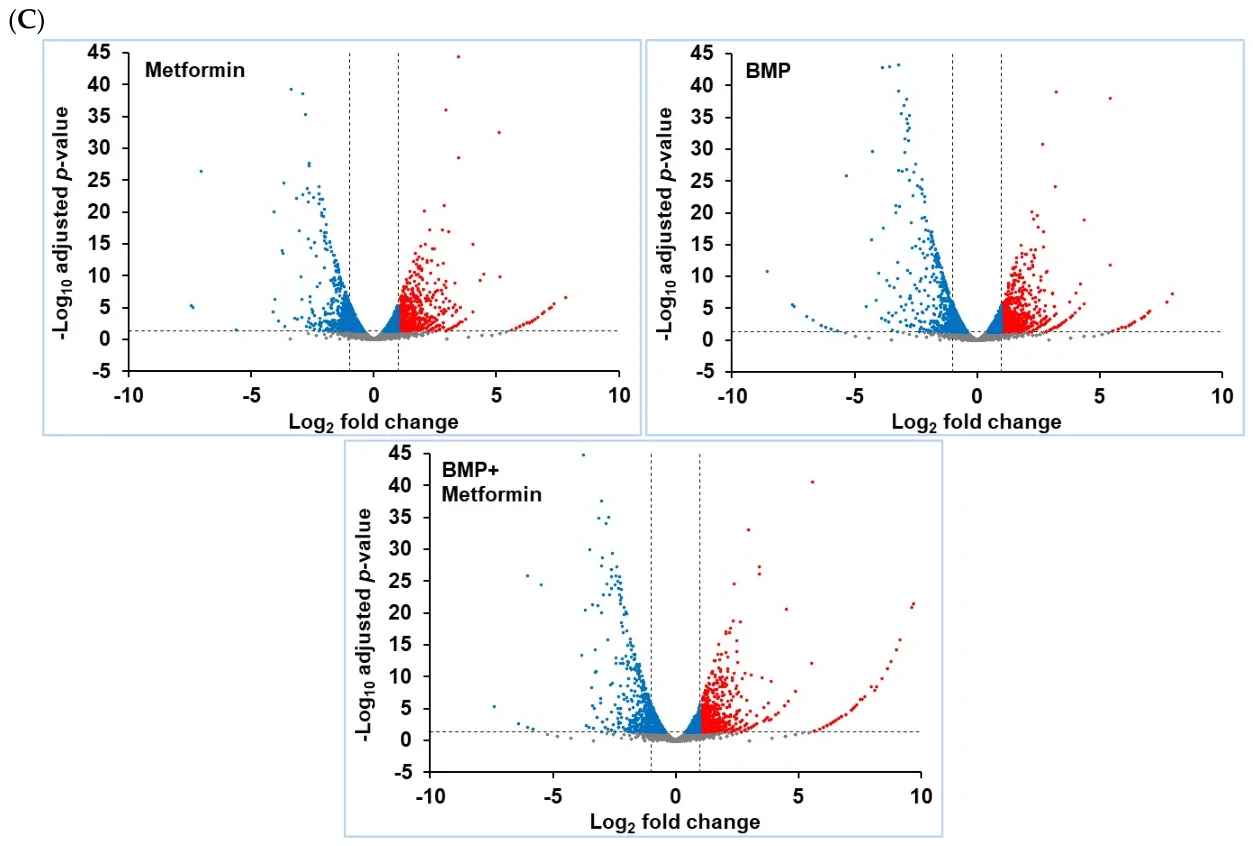
Effects of BMP and metformin on physiological parameters and skeletal muscle transcriptomic responses in *db*/*db* mice. (**A**) Weekly body weight and average food intake over the 8-week treatment period. (**B**) Fasting blood glucose levels and HbA1c percentages measured at weeks 0, 4, and 8. Data are presented as mean ± SD (n = 7 mice/group). Between-group differences at each indicated time point were evaluated using one-way ANOVA followed by post-hoc Bonferroni pairwise comparisons. * *p* < 0.05, *** *p* < 0.001, compared to the mock group; # *p* < 0.05, ## *p* < 0.01, compared to the metformin group at indicated time points. (**C**) Volcano plots illustrating global transcriptomic changes in skeletal muscle from *db*/*db* mice treated with metformin, BMP, or their combination. Red dots indicate significantly upregulated genes (log_2_ fold change ≥ 1, adjusted *p* < 0.05), and blue dots indicate significantly downregulated genes (log_2_ fold change ≤ −1, adjusted *p* < 0.05). Gray dots represent non-significant transcripts. Dashed lines indicate the thresholds for statistical significance and fold change.

To investigate the effects of BMP alone or in combination with metformin on glycemic control, *db/db* mice were orally administered BMP, metformin, or their combination daily. Throughout the study, no significant differences in body weight or daily food intake were observed among groups (Figure 2A), suggesting that the glucose-lowering effects were independent of changes in caloric intake or body weight. Fasting blood glucose and HbA1c levels were measured at weeks 4 and 8. As shown in Figure 2B, fasting blood glucose levels in the mock group remained around 500 mg/dL. By week 8, BMP treatment reduced glucose levels by approximately 200 mg/dL compared to the mock group, while the combination of BMP and metformin reduced fasting blood glucose to ~220 mg/dL, representing a ~310 mg/dL lower level compared to the mock group (*p* < 0.001 vs. mock; *p* < 0.05 vs. metformin alone). No hypoglycemic events were observed in any treatment group. Similarly, HbA1c levels in the mock group increased from 11.23 ± 1.66% to 13.4 ± 1.09% over 8 weeks. In contrast, HbA1c levels were maintained in the metformin group, decreased by approximately 1% in the BMP group, and decreased by approximately 2% in the BMP + metformin group (*p* < 0.001 vs. mock; *p* < 0.05 vs. metformin alone). Collectively, these findings indicate that BMP alone improves glycemic control, and its combination with metformin results in a greater reduction in both fasting blood glucose and HbA1c levels.

To further evaluate the effects of combination treatment at the molecular level, transcriptomic profiling of skeletal muscle was performed using RNA-Seq. The BMP + metformin group exhibited a markedly greater number of DEGs compared with either BMP or metformin alone (Figure 2C). Specifically, the number of genes with a log_2_ fold change > 1 (adjusted *p* < 0.05) was substantially higher in the combination group. Direct contrast analysis comparing the combination treatment to each monotherapy group revealed 105 genes that were uniquely regulated by the combination therapy (fold change ≥ 2 or ≤ −2, adjusted *p* < 0.05). Rather than merely reflecting overall gene counts, these non-additive transcriptomic alterations highlight candidate signaling pathways modulated exclusively under combined administration.

### 3.3. BMP and Metformin Converge on Shared Skeletal Muscle Metabolic Pathways

**Figure 3:**
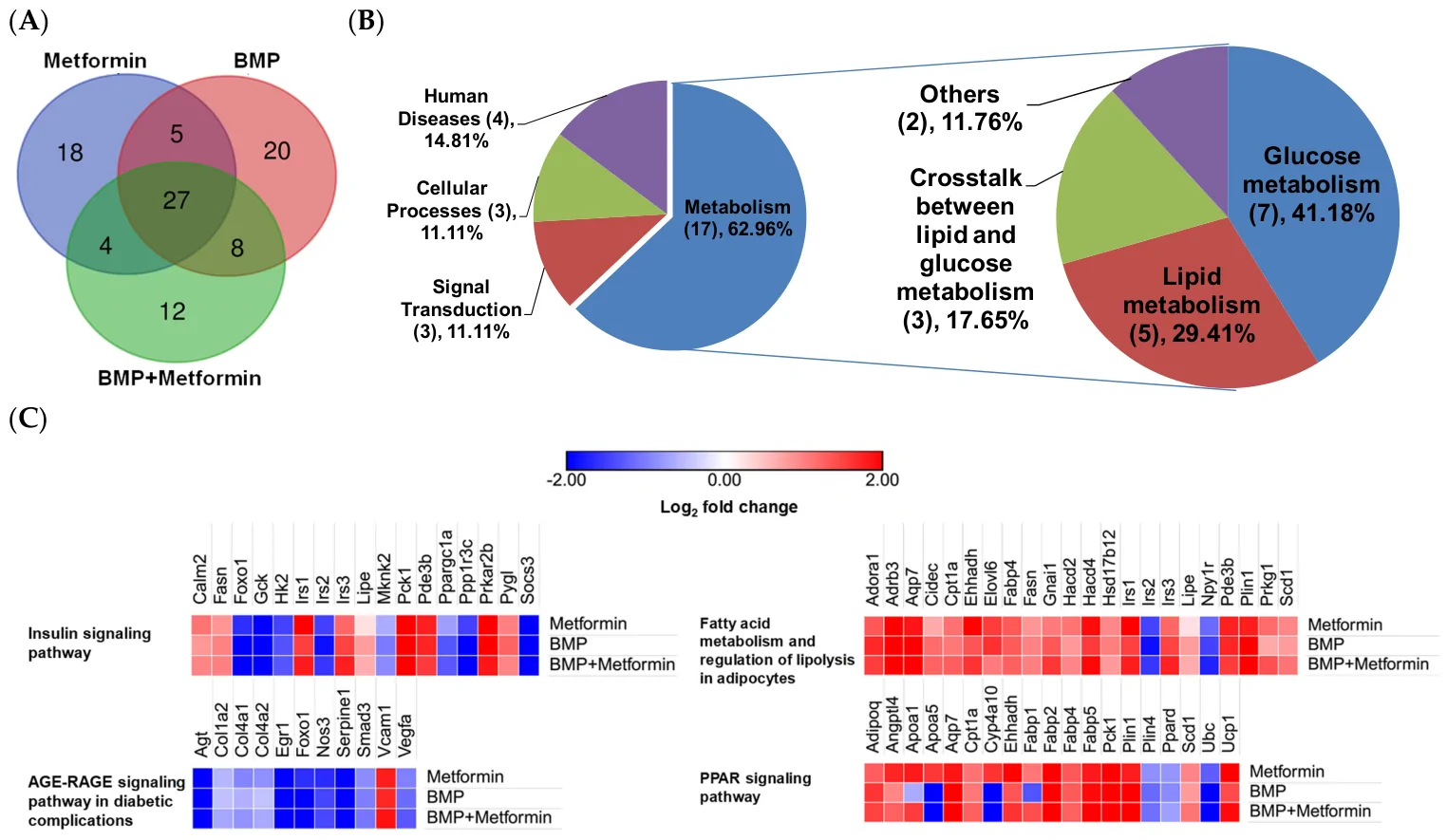
KEGG pathway enrichment analysis of DEGs in skeletal muscle following BMP and metformin treatment. (**A**) Venn diagram showing the number of significantly enriched KEGG pathways in BMP, metformin, and BMP + metformin groups, and the overlap of commonly regulated pathways. (**B**) Functional classification of the 27 commonly enriched KEGG pathways into four major categories: metabolism, signal transduction, cellular processes, and human diseases. The right panel shows a detailed subclassification of metabolism-related pathways. (**C**) Heatmap showing the expression patterns of DEGs involved in representative metabolic pathways, including insulin signaling pathway, AGE-RAGE signaling pathway in diabetic complications, fatty acid metabolism and regulation of lipolysis in adipocytes, and PPAR signaling pathways. Color scale represents normalized gene expression levels (log_2_ fold change), with red indicating upregulation and blue indicating downregulation.

To further elucidate the biological pathways affected by BMP alone or in combination with metformin, DEGs were subjected to KEGG pathway enrichment analysis. As shown in Figure 3A, a total of 60, 54, and 51 pathways were significantly enriched in the BMP, metformin, and BMP + metformin groups, respectively. Venn diagram analysis revealed 27 pathways that were commonly regulated across all three treatment groups (Figure 3A and Table 1). These shared pathways were further categorized into four major functional groups, including metabolism, signal transduction, cellular processes, and human diseases (Figure 3B). Among these categories, metabolism-related pathways predominated, accounting for 17 of the 27 common pathways (62.96%). Notably, most of these metabolic pathways were directly associated with glucose and lipid metabolism, including insulin signaling pathway, insulin resistance, T2DM, fatty acid metabolism, regulation of lipolysis in adipocytes, and peroxisome proliferator-activated receptor (PPAR) signaling pathway.

Consistent with this classification, several key metabolic pathways exhibited strong enrichment across treatment groups (Table 1). In particular, the PPAR signaling pathway showed highly significant enrichment (adjusted *p* = 1.66 × 10^−10^ in metformin; 6.64 × 10^−9^ in BMP), along with the AMP-activated protein kinase (AMPK) signaling pathway (adjusted *p* = 2.03 × 10^−8^ in BMP) and the phosphatidylinositol 3′-kinase (PI3K)-Akt signaling pathway (adjusted *p* = 1.33 × 10^−9^ in BMP). These pathways represent central regulatory hubs that integrate glucose utilization, lipid metabolism, and insulin signaling. In addition, enrichment of the forkhead box O (FoxO) and hypoxia-inducible factor-1 (HIF-1) signaling pathways suggests the involvement of oxidative stress responses and metabolic adaptation. Furthermore, the inclusion of the receptor for advanced glycation end-products (RAGE) signaling pathway in diabetic complications indicates that BMP and metformin may modulate inflammation- and oxidative stress-related processes associated with insulin resistance.

To visualize the transcriptional regulation within these pathways, Figure 3C presents the expression patterns of DEGs involved in representative metabolic pathways. The heatmap demonstrates that the majority of genes exhibited consistent directional changes across BMP, metformin, and BMP + metformin groups, indicating a high degree of overlap in their regulatory effects. Notably, the combination treatment did not uniformly increase enrichment magnitude but instead showed a coordinated modulation pattern across these pathways. Taken together, these data indicate that BMP and metformin converge on a shared network of metabolic signaling pathways in skeletal muscle, particularly those centered on AMPK, PI3K-Akt, PPAR, and FoxO signaling. Rather than demonstrating formal pharmacological synergy, this coordinated regulation reflects biological pathway convergence, providing a mechanistic basis for the observed improvements in metabolic parameters and supporting the role of BMP as a complementary intervention to metformin.

### 3.4. Participant Enrollment and Baseline Characteristics

**Figure 4:**
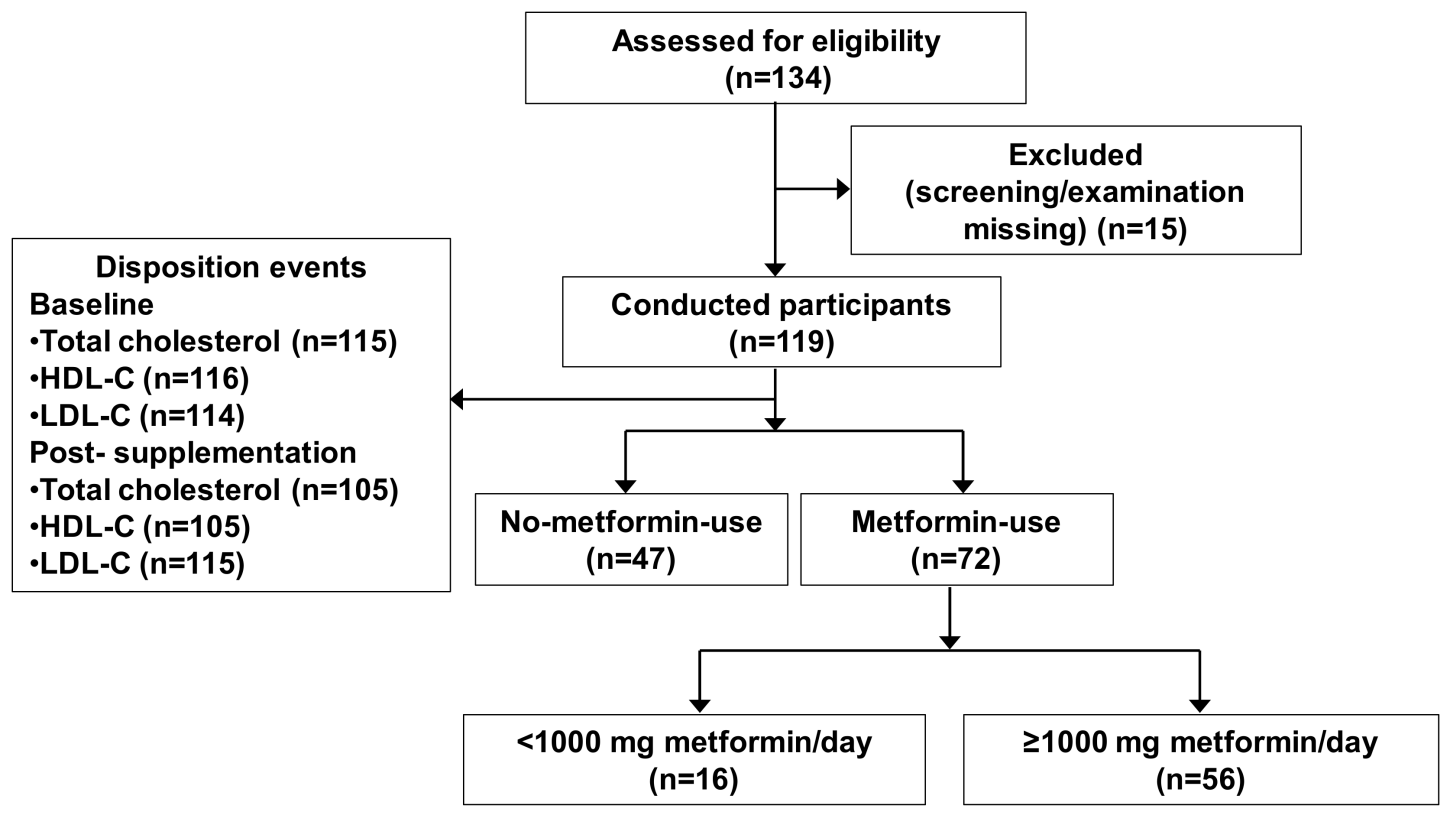
Flow diagram of participant enrollment and grouping in the self-controlled retrospective cohort study.

To complement the mechanistic findings from the T2DM mouse model, a translational approach was employed by integrating real-world clinical data from a self-controlled retrospective cohort. As shown in Figure 4, a total of 134 participants with prediabetes or diabetes who continuously used BMP for three months were initially enrolled. Among them, 15 participants who did not complete at least two follow-up visits within one year were excluded, resulting in a final cohort of 119 participants. The study population was stratified into a no-metformin-use group (n = 47) and a metformin-use group (n = 72). The metformin-use group was further subdivided into participants receiving <1000 mg/day (n = 16) and ≥1000 mg/day (n = 56) of metformin. The cutoff of 1000 mg/day was chosen based on clinical practice guidelines and pharmacological dosing thresholds for metformin. In clinical management of T2DM, 1000 mg/day represents the recognized transition threshold between initial low/titration dosing and optimal target therapeutic maintenance dosing [3,4].

Baseline characteristics of the study population are summarized in Table 2. The overall cohort had a mean age of 63.03 ± 12.25 years and a mean BMI of 26.45 ± 4.47 kg/m^2^. Most of the participants were female (65.55%). No significant differences were observed between the no-metformin-use and metformin-use groups in terms of age, sex distribution, anthropometric parameters (height, weight, and BMI), or diabetes duration. As expected, differences in clinical composition were observed between groups. The no-metformin-use group included participants with prediabetes (61.7%) and a small proportion with T1DM (4.26%), whereas all participants in the metformin-use group were diagnosed with T2DM. Fasting glucose levels were higher in the metformin-use group compared to the no-metformin-use group, reflecting differences in disease status at baseline. Within the metformin-use group, baseline characteristics were generally comparable between the <1000 mg/day and ≥1000 mg/day subgroups. To reflect real-world clinical practice where dietary supplements are taken across a spectrum of metabolic conditions, all eligible participants, including those with prediabetes, T1DM (n = 2), and T2DM (n = 88), were retained in the primary observational analysis. In addition, real-world clinical data were obtained from electronic medical records. Missing laboratory measurements at specific follow-up time points were treated as missing at random and analyzed using an available-case framework without artificial data imputation.

### 3.5. Effects of BMP on Serum Glycemic and Lipid Indices

Based on transcriptomic findings indicating that BMP modulates pathways governing glucose and lipid metabolism, we evaluated the clinical effects of BMP supplementation on glycemic and lipid parameters in a real-world cohort (N = 119). Baseline HbA1c was higher among metformin users (8.1 ± 1.2%) than non-users (6.4 ± 0.8%), reflecting routine clinical treatment indications. Within-subject longitudinal evaluations demonstrated distinct glycemic trajectories across medication subgroups following BMP supplementation.

Following 12 weeks of BMP supplementation, a significant reduction in HbA1c was observed in background metformin users (Δ = −0.23%, *p* = 0.0028), whereas non-users showed no significant change (Δ = +0.02%, *p* = 0.8174; Table 3). Similarly, significant longitudinal reductions were observed among metformin users for total cholesterol (Δ = −7.5 mg/dL, *p* = 0.0331) and LDL-C (Δ = −6.1 mg/dL, *p* = 0.0021), with no significant changes in non-users (Table 4 and Table 5). Because clinical data were derived from real-world routine outpatient records, available sample sizes for individual lipid parameters fluctuated across evaluation time points (−3, 0, and +3 months) based on complete-case laboratory availability. In the non-metformin subgroup, post-supplementation total cholesterol exhibited a minor mean increase of 1.9 mg/dL, which achieved statistical significance (*p* = 0.0213) primarily owing to low intra-individual variability across complete paired measurements (n = 105). HDL-C levels remained unchanged across groups, exhibiting substantial inter-individual variability (Table 6). Post-supplementation HDL-C standard deviations (50.8 and 57.6 mg/dL) reflect baseline clinical heterogeneity and the presence of a small subset of extreme high-HDL outliers in the retrospective cohort. Sensitivity analysis excluding these outliers yielded equivalent results without altering the significance of primary outcomes.

A pre-planned sensitivity analysis restricting the dataset strictly to the homogeneous T2DM population (n = 88, excluding prediabetes and n = 2 T1DM) yielded highly consistent findings, with metformin users maintaining a significant adjusted HbA1c reduction (Δ = −0.24%, *p* = 0.0021). This dual analytical approach confirms that BMP supplementation is associated with glycemic and lipid improvements in background metformin users, and that these observations are robust and independent of baseline glycemic severity or sample heterogeneity.

### 3.6. Associated Changes in HbA1c Profiles in Participants Stratified by Metformin Treatment

**Figure 5:**
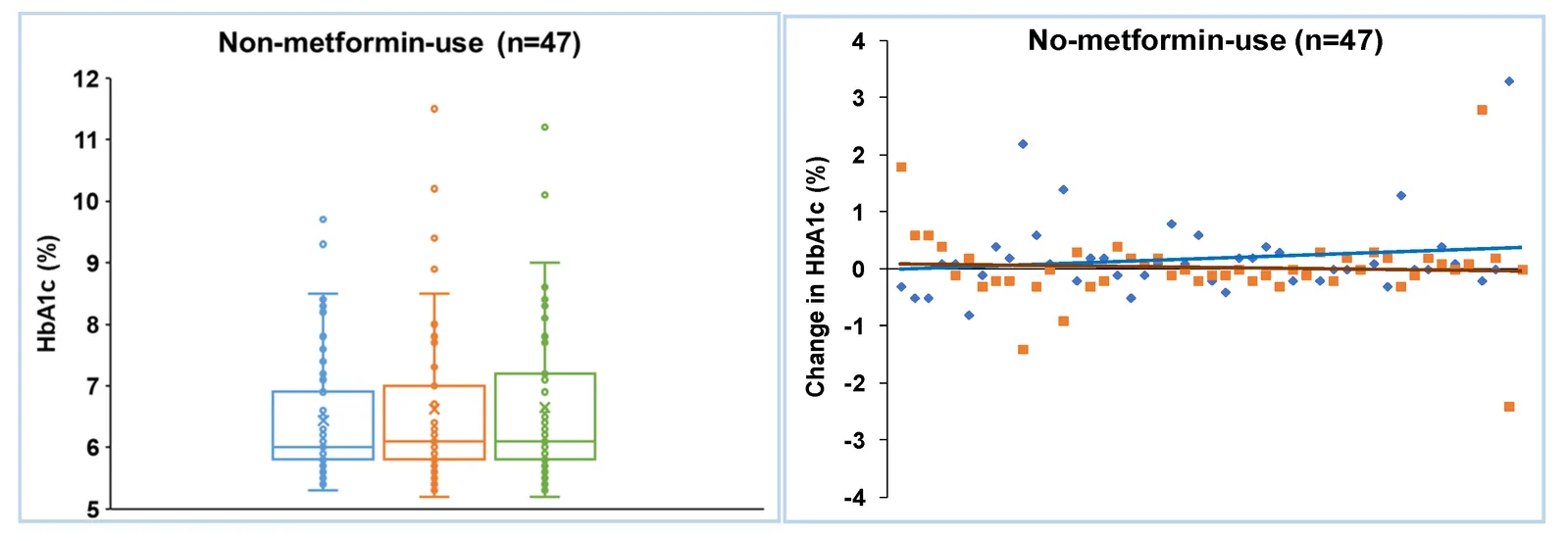

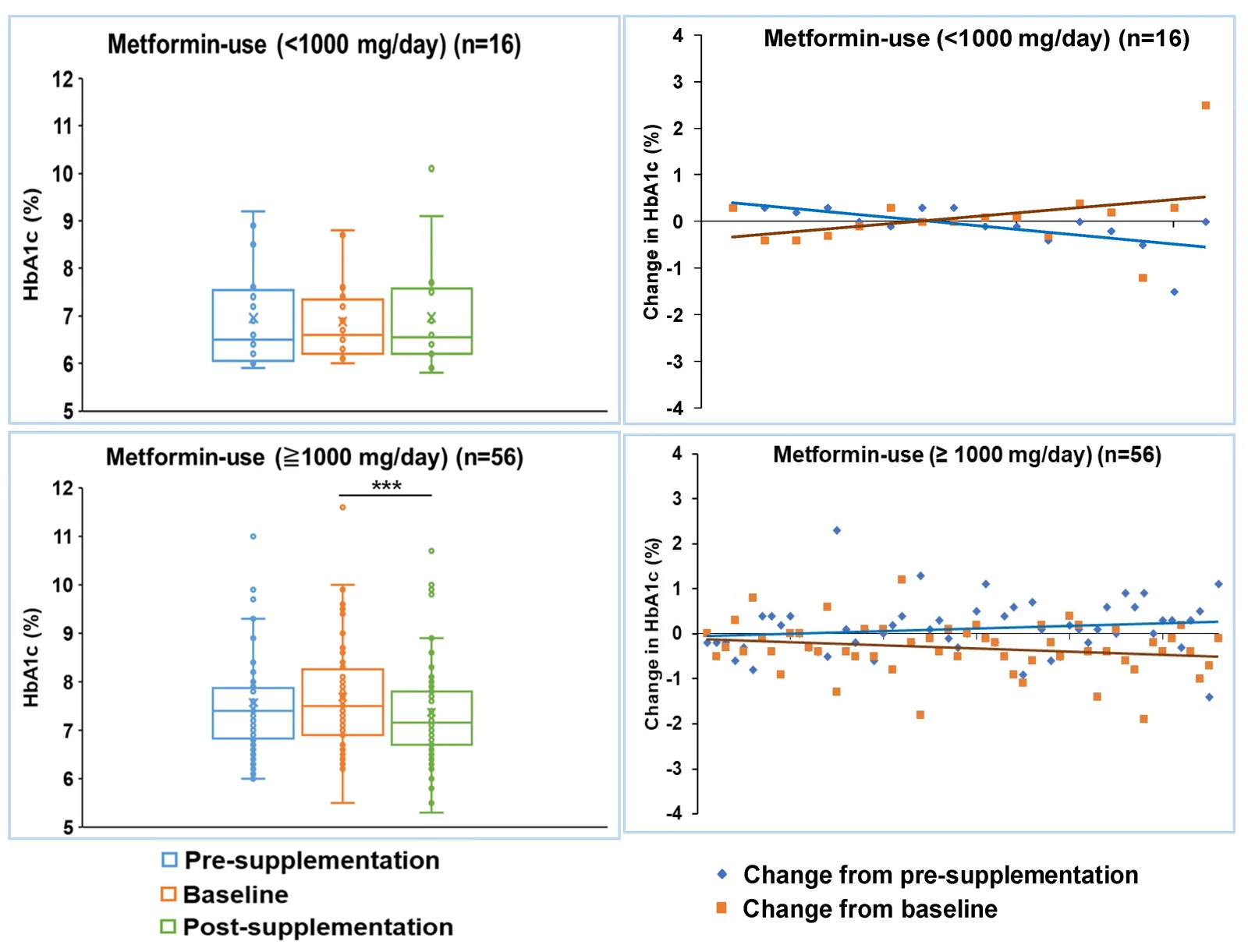
Longitudinal changes in HbA1c levels following BMP supplementation in participants with or without metformin treatment. HbA1c levels (%) were assessed at pre-supplementation (−3 months), baseline (0 month), and post-supplementation (+3 months). Participants were stratified into no-metformin-use (n = 47) and metformin-use groups, with the latter further subdivided into <1000 mg/day (n = 16) and ≥1000 mg/day (n = 56) subgroups. The left panel shows the distribution of HbA1c levels at each time point. Boxes represent the interquartile range, the horizontal line indicates the median, “×” denotes the mean, and whiskers extend to the minimum and maximum values excluding outliers. The right panel illustrates individual changes in HbA1c. Each dot represents the change in HbA1c for an individual participant, with blue dots indicating changes from pre-supplementation to baseline and orange dots indicating changes from baseline to post-supplementation. The line represents the overall trend of these changes. *** *p* < 0.001, compared to the baseline.

To evaluate whether BMP supplementation is associated with differential glycemic outcomes across background therapies, participants were stratified according to metformin use and daily dosage (Table 3 and Figure 5). In the no-metformin-use group, HbA1c levels showed a gradual increase over time, rising from 6.43% at pre-supplementation to 6.62% at baseline and 6.64% at post-supplementation. However, these changes were not statistically significant (Δ = +0.02%, 95% CI: [−0.15, 0.19], *p* = 0.8174, Cohen’s *d* = 0.01), indicating that BMP alone was not associated with significant HbA1c reductions in this subgroup. In contrast, in the metformin-use group, which exhibited higher baseline HbA1c levels (7.51%), HbA1c slightly increased from pre-supplementation (7.43%) to baseline (7.51%), followed by a significant decrease to 7.28% after BMP supplementation (Δ = −0.23%, 95% CI: [−0.37, −0.09], *p* = 0.0028, Cohen’s *d* = 0.38). This observation suggests that BMP supplementation is primarily associated with glycemic improvements among participants receiving concurrent metformin.

To further assess trends across background medication levels, the metformin-use group was stratified by daily dosage. In participants receiving <1000 mg/day, HbA1c levels remained stable over time, with no significant changes observed (Table 3 and Figure 5). In contrast, participants receiving ≥1000 mg/day exhibited a significant reduction in HbA1c from baseline (7.69%) to post-supplementation (7.37%), corresponding to a decrease of −0.32% (95% CI: [−0.45, −0.19], *p* < 0.0001, Cohen’s *d* = 0.54). These findings indicate that the HbA1c reduction observed during BMP supplementation was most prominent in individuals receiving higher background doses of metformin, representing a dose-associated clinical trend.

## 4. Discussion

Dietary interventions and nutraceuticals are increasingly investigated as adjuncts to standard pharmacotherapy for improving glycemic control and cardiometabolic risk in T2DM [5,6,7,8,9,10]. Micronutrients, plant-derived bioactives, and fiber-rich foods exert modest but clinically meaningful effects on glucose homeostasis and lipid metabolism. Representative examples include chromium supplementation (250 µg/day), which improves fasting glucose and HbA1c [34], and dietary patterns such as the ketogenic diet, which reduce fasting glucose and HbA1c while improving lipid profiles [35]. Bioactives such as ginsenosides enhance insulin secretion and sensitivity, lowering fasting glucose, total cholesterol, and the Homeostasis Model Assessment of Insulin Resistance [36], whereas cinnamon (*Cinnamomum cassia*) improves fasting glucose [37,38]. Fiber-rich foods, including oats (*Avena sativa* L.) and β-glucan-rich barley, attenuate postprandial glycemic excursions and modulate hormonal responses [14,39]. Emerging evidence further supports beneficial metabolic effects of mango, red dragon fruit, and kale supplementation [12,13,16]. However, heterogeneity in efficacy remains a consistent limitation across studies, often reflecting differences in baseline metabolic status, study design, and concurrent medication use. Within this context, identifying nutraceuticals that complement first-line therapies rather than replace them represents a key translational priority.

Among botanical candidates, bitter melon has garnered attention for its antidiabetic properties [20,40]. Its bioactivity has been attributed to multiple components capable of activating PPAR pathways, upregulating glucose transporter type 4 (GLUT4), and modulating incretin-related signaling [24]. In particular, bitter melon-derived peptides, including BMP and its gastro-resistant derivatives, have been shown to directly engage the IR and activate downstream signaling intermediates such as PI3K and Akt, thereby promoting GLUT4-mediated glucose uptake [25,26,27,28,29]. Preclinical studies further demonstrate reductions in blood glucose and HbA1c, improved survival, and attenuation of diabetic complications following long-term administration [29,41,42]. These data support the biological plausibility of bitter melon-derived peptides as nutraceutical adjuncts targeting insulin signaling pathways.

In the present study, we combined in vitro digestion, peptidomics, transcriptomics, and real-world clinical data to evaluate the metabolic effects of a BMP-enriched preparation. Across these complementary approaches, our findings converge on a model in which BMP functions as a metformin-dependent metabolic modulator, with measurable benefits observed primarily in individuals receiving metformin and in those with greater baseline metabolic dysregulation. From a biochemical perspective, simulated gastrointestinal digestion of BMP generated a peptide pool enriched in short-chain fragments (5–10 amino acids), a size range generally associated with improved intestinal absorption and bioactivity. The high abundance of IVARPPTIG, a peptide previously reported to interact with the IR [25], supports the retention of functional motifs following digestion, an essential consideration for translational applicability. The relatively low peptide identification rate observed in the peptidomic dataset is consistent with the known analytical challenges of plant-derived peptide mixtures and likely reflects database annotation limitations rather than incomplete digestion.

At the organismal level, BMP improved glycemic control in *db*/*db* mice, with greater effects observed under co-administration with metformin, and without detectable changes in body weight or food intake. This dissociation suggests a primary metabolic rather than behavioral mechanism. Transcriptomic profiling of skeletal muscle further revealed that combination treatment induced broader gene expression changes than either intervention alone, particularly within pathways related to energy metabolism. Enrichment analyses consistently identified pathways that regulate glucose uptake, lipid utilization, and insulin responsiveness, thereby providing a coherent mechanistic framework linking molecular signaling to systemic metabolic outcomes. Importantly, the observed pathway convergence indicates that BMP likely reinforces existing metabolic networks rather than introducing novel signaling axes. However, while transcriptomic profiling demonstrated widespread alterations in the combination group, an expanded pool of DEGs does not inherently denote superior therapeutic efficacy; rather, these shifts represent candidate networks perturbed by dual treatment. Consequently, detailed downstream functional validation, specifically at the protein phosphorylation and post-translational signaling levels, is required to definitively establish causality and identify true driver pathways.

Consistent with these mechanistic insights, the clinical data demonstrated that BMP supplementation was associated with improvements in HbA1c, total cholesterol, and LDL-C predominantly in participants receiving metformin, whereas no significant effects were detected in those not treated with metformin. Furthermore, the magnitude of HbA1c reduction was greater among individuals receiving 1000 mg/day of metformin. However, this pattern must be interpreted cautiously. The metformin-treated group consisted of T2DM patients with higher baseline HbA1c levels, whereas non-users included prediabetic individuals with lower baseline values. Thus, the greater reductions observed in metformin users may reflect underlying baseline disease severity and regression to the mean rather than true biological synergy or a definitive pharmacodynamic interaction.

This mechanistic overlap remains biologically plausible. Metformin primarily acts via AMPK activation to suppress hepatic gluconeogenesis and improve peripheral sensitivity [3,43], whereas BMP and its active peptides enhance IR autophosphorylation [25,26,27,28,29]. The overlapping transcriptomic profiles observed in skeletal muscle highlight a convergence on downstream hubs (such as PI3K-Akt and PPAR pathways). Rather than demonstrating formal drug–drug synergy, these data suggest that BMP and metformin exert complementary effects by simultaneously engaging distinct entry points within common metabolic networks.

Compared with prior clinical studies, which have largely evaluated bitter melon as a monotherapy and reported variable outcomes, the present work offers a distinct perspective on its potential as an adjunctive intervention. Previous trials of bitter melon extracts or peptide formulations have demonstrated reductions in fasting and postprandial glucose, though with inconsistent effects on HbA1c (typically yielding mean reductions ranging from −0.24% to −0.50%) [18,19,20,21], with select formulations showing efficacy comparable to metformin [44]. In our real-world clinical cohort of metformin-treated individuals, BMP supplementation was associated with a statistically significant mean HbA1c reduction of −0.23% (reaching −0.32% in responsive sub-analyses) alongside a minor LDL-C decrease of approximately 6 mg/dL. When evaluating these findings, it is essential to distinguish statistical significance from clinical meaningfulness. While modest compared to second-line pharmacological agents, such as glucagon-like peptide-1 receptor agonists (−1.0% to −1.8%) [45] or sodium-glucose co-transporter 2 inhibitors (−0.6% to −1.0%) [46], this −0.23% to −0.32% reduction falls squarely within the expected range for dietary supplements and lifestyle modifications (0.2% to 0.6%). Thus, rather than driving dramatic disease modification or potent pharmacological synergy, BMP may function as a modest pharmaconutritional adjunct alongside first-line therapy to provide complementary metabolic support. Nevertheless, given the non-randomized, retrospective nature of this study, these exploratory outcomes warrant caution and require prospective validation.

Several limitations of this study warrant consideration. First, as a real-world retrospective analysis lacking randomization or a concurrent placebo-controlled arm, these clinical findings represent observational associations rather than confirmed therapeutic causality. Second, the primary cohort (N = 119) encompassed baseline heterogeneity across clinical indications, including small subsets with prediabetes and T1DM (n = 2); nevertheless, multivariable statistical adjustments and restricted T2DM sensitivity analyses confirmed the stability of our primary outcomes. Third, owing to the boundaries of retrospective electronic health record extractions, detailed historical parameters, such as exact diabetes duration, continuous longitudinal medication titrations, quantitative dietary assessments, physical activity, and body weight fluctuations, were not systematically recorded, leaving potential residual confounding by unmeasured non-antidiabetic factors. Specifically, while patients maintained stable baseline regimens per routine clinical care, granular dose-level trajectories for non-primary antidiabetic medications could not be extracted, and metformin dosing was analyzed categorically (<1000 mg/day vs. ≥1000 mg/day) rather than continuously due to retrospective clustering around standard clinical dosage steps. Fourth, treatment adherence and safety tracking relied on routine outpatient follow-up consultations, prescription refill records, and standard clinical laboratory monitoring (liver and renal function panels); consequently, formal prospective adherence measures were not implemented, and mild, self-limiting adverse events or non-eventful hypoglycemic episodes not brought to clinical attention could not be systematically captured. Finally, the small sample size of certain sub-cohorts (n = 16 in the non-metformin subgroup) limits statistical power, requiring subgroup observations to be interpreted as exploratory. Prospective, double-blind, randomized controlled trials in defined clinical populations with comprehensive lifestyle, adherence, and medication tracking are needed to confirm these preliminary findings.

## 5. Conclusions

In conclusion, this study provides a translational framework evaluating the metabolic effects of BMP alongside background metformin therapy. To our knowledge, this work is the first to demonstrate that BMP, a major protein component of bitter melon, exhibits complementary glucose-lowering effects and coordinated skeletal muscle pathway remodeling when combined with first-line antidiabetic therapy in preclinical models, while demonstrating a corresponding observational association in a real-world clinical setting. In the clinical cohort, BMP supplementation was associated with modest reductions in HbA1c and lipid parameters primarily among participants receiving concurrent metformin, particularly at standard therapeutic dosages (≥1000 mg/day). However, given the retrospective self-controlled design and baseline clinical heterogeneity between treatment subgroups, these clinical findings reflect observational associations rather than confirmed causality or proven drug–supplement synergy. Overall, these findings highlight BMP as a promising complementary candidate in metabolic management and establish an important foundation for nutritional protein research. Prospective, randomized, double-blind controlled trials with standardized dosing, matched cohorts, and prespecified interaction analyses are required to confirm these exploratory observations and definitively establish the role of BMP as a pharmaconutritional adjunct in diabetes care.

## Figures and Tables

**Table 1:** KEGG pathway enrichment analysis of pathways commonly regulated by BMP, metformin, and their combination in skeletal muscle of *db*/*db* mice.

Category	ID	Description	Metformin		BMP		BMP + Metformin	
			Count ^a^	Adjusted *p*-Value	Count	Adjusted *p*-Value	Count	Adjusted *p*-Value
Glucose metabolism	mmu04910	Insulin signaling pathway	16	0.0166	22	2.16 × 10^−5^	17	0.0009
	mmu04931	Insulin resistance	17	0.0006	19	2.24 × 10^−5^	13	0.0048
	mmu04930	Type II diabetes mellitus	8	0.0107	10	0.0004	7	0.0117
	mmu04068	FoxO signaling pathway	16	0.0097	20	8.55 × 10^−5^	17	0.0005
	mmu04933	AGE-RAGE signaling pathway in diabetic complications	14	0.0051	14	0.0025	11	0.0162
	mmu04066	HIF-1 signaling pathway	22	2.76 × 10^−6^	19	3.75 × 10^−5^	13	0.0064
	mmu04010	MAPK signaling pathway	31	0.0044	43	2.89 × 10^−8^	34	1.02 × 10^−5^
Lipid metabolism	mmu01212	Fatty acid metabolism	14	2.9 × 10^−5^	9	0.0105	8	0.0149
	mmu01040	Biosynthesis of unsaturated fatty acids	8	0.0011	5	0.0487	5	0.0305
	mmu04923	Regulation of lipolysis in adipocytes	13	4.99 × 10^−5^	16	1.16 × 10^−7^	14	9.19 × 10^−7^
	mmu03320	PPAR signaling pathway	25	1.66 × 10^−10^	22	6.64 × 10^−9^	19	1.18 × 10^−7^
	mmu04920	Adipocytokine signaling pathway	11	0.0053	11	0.0029	10	0.0037
Crosstalk between lipid and glucose metabolism	mmu04152	AMPK signaling pathway	20	0.0002	26	2.03 × 10^−8^	18	0.0001
	mmu04151	PI3K-Akt signaling pathway	47	2.03 × 10^−6^	52	1.33 × 10^−9^	41	1.69 × 10^−6^
	mmu04211	Longevity regulating pathway	11	0.0289	14	0.0008	9	0.0452
	mmu00910	Nitrogen metabolism	4	0.0206	7	2.91 × 10^−5^	5	0.0014
	mmu04964	Proximal tubule bicarbonate reclamation	7	0.0003	6	0.0014	7	8.82 × 10^−5^
Signal Transduction	mmu04371	Apelin signaling pathway	23	1.87 × 10^−5^	24	1.47 × 10^−6^	23	5.79 × 10^−7^
	mmu04015	Rap1 signaling pathway	27	0.0006	28	6.41 × 10^−5^	23	0.0008
	mmu04115	p53 signaling pathway	10	0.0163	12	0.0009	10	0.0040
Cellular Processes	mmu04210	Apoptosis	15	0.0281	14	0.0311	15	0.0049
	mmu04710	Circadian rhythm	7	0.0052	6	0.0139	5	0.0305
	mmu04510	Focal adhesion	25	0.0011	24	0.0008	17	0.0343
Human Diseases	mmu05414	Dilated cardiomyopathy	15	2.47 × 10^−5^	14	0.0012	10	0.0247
	mmu01521	EGFR tyrosine kinase inhibitor resistance	10	0.0294	14	0.0002	9	0.0217
	mmu05418	Fluid shear stress and atherosclerosis	20	0.0011	22	5.22 × 10^−5^	19	0.0002
	mmu05410	Hypertrophic cardiomyopathy	17	5.74 × 10^−5^	13	0.0027	10	0.0201

^a^ Count indicates the number of DEGs mapped to each pathway.

**Table 2:** Baseline characteristics of the study participants.

Variable ^a^	Overall (n = 119)	No-Metformin-Use (n = 47)	Metformin-Use		
			Overall (n = 72)	<1000 mg/day (n = 16)	≥1000 mg/day (n = 56)
Age (y)	63.03 ± 12.25	62.64 ± 13.93	63.28 ± 11.02	62.75 ± 7.59	63.43 ± 11.53
Female, n (%)	78 (65.55%)	32 (68.09%)	46 (63.89%)	11 (68.75%)	35 (62.5%)
Male, n (%)	41 (34.45%)	15(31.91%)	26 (36.11%)	5 (31.25%)	21 (37.5%)
Height (cm)	159.35 ± 8.41	158.48 ± 7.31	159.91 ± 9.06	158.36 ± 7.98	160.76 ± 9.07
Weight (kg)	67.47 ± 13.98	66.85 ± 15.79	67.87 ± 12.77	64.38 ± 8.71	68.89 ± 14.24
BMI (kg/m^2^)	26.45 ± 4.47	26.46 ± 5.27	26.45 ± 3.90	25.67 ± 2.67	26.61 ± 4.49
Glucose (AC) (mg/dL)	135.36 ± 37.57	114.70 ± 24.88	148.85 ± 38.44	131.59 ± 33.85	134.94 ± 37.92
Diabetes subtype					
Pre-diabetes, n (%)	29 (24.37%)	29 (61.7%)	0	0	0
Type 1, n (%)	2 (1.68%)	2 (4.26%)	0	0	0
Type 2, n (%)	88 (73.95%)	16 (34.04%)	72 (100%)	16 (100%)	56 (100%)
Diabetes duration (y) ^b^	14.7 ± 8.91	10.67 ± 11.47	14.51 ± 8.02	12.19 ± 7.68	15.18 ± 8.06

^a^ Data are expressed as mean ± SD or n (%). ^b^ Diabetes duration (years) was calculated for participants diagnosed with diabetes.

**Table 3:** Effects of BMP on HbA1c levels (%) in participants with or without metformin treatment.

Group	Case No.	Pre-Supplementation ^a^	Baseline ^a^	Post-Supplementation ^a^	Change from Pre-Supplementation ^b^	*p* Value ^c^	Change from Baseline ^b^	*p* Value ^c^
No-metformin-use	47	6.43 ± 1.07	6.62 ± 1.37	6.64 ± 1.31	0.19	0.0693	0.02	0.8174
Metformin-use								
Overall	72	7.43 ± 1.15	7.51 ± 1.14	7.28 ± 1.11	0.08	0.2860	−0.23	0.0028 **
<1000 mg/day	16	6.96 ± 1.09	6.88 ± 0.88	6.98 ± 1.19	−0.08	0.5213	0.09	0.6266
≥1000 mg/day	56	7.57 ± 1.14	7.69 ± 1.15	7.37 ± 1.08	0.12	0.1565	−0.32	<0.0001 ***

^a^ Data are expressed as mean ± SD. ^b^ Changes from pre-supplementation (−3 months) and baseline (0 month) represent absolute mean differences (Δ). ^c^ Longitudinal changes were evaluated using paired *t*-tests under a complete-case analysis approach. ** *p* < 0.01, *** *p* < 0.001, compared to the baseline.

**Table 4:** Effects of BMP on total cholesterol (mg/dL) in participants with or without metformin treatment.

Group	Case No.	Pre-Supplementation ^a^	Case No.	Baseline	Case No.	Post-Supplementation ^a^	Change from pre-Supplementation ^b^	*p* Value ^c^	Change from Baseline ^b^	*p* Value ^c^
No-metformin-use	47	177.1 ± 39.8	44	169.6 ± 35.7	38	171.5 ± 38.1	−7.5	0.2241	1.9	0.0213 *
Metformin-use										
Overall	72	151.1 ± 30.1	71	152.2 ± 30.9	67	144.7 ± 29.5	1.1	0.6629	−7.5	0.0331 *
<1000 mg/day	16	160.1 ± 25.6	16	162.6 ± 26.1	15	158.9 ± 23.5	2.5	0.3523	−3.7	0.5336
≥1000 mg/day	56	148.4 ± 31	55	149.2 ± 31.7	52	140.5 ± 30	0.8	0.8196	−8.7	0.0425 *

^a^ Data are expressed as mean ± SD for available participants at each specified time point. ^b^ Changes from pre-supplementation (−3 months) and baseline (0 month) represent absolute mean differences (Δ). ^c^ *p*-values were calculated using paired *t*-tests with Bonferroni correction for multiple comparisons to evaluate differences between pre-supplementation (−3 months) and baseline (0 month), as well as baseline (0 month) and post-supplementation (+3 months). Sample sizes at each time point reflect complete paired data available from routine clinical testing. * *p* < 0.05, compared to the baseline.

**Table 5:** Effects of BMP on LDL-C (mg/dL) in participants with or without metformin treatment.

Group	Case No.	Pre-Supplementation ^a^	Case No.	Baseline	Case No.	Post-Supplementation ^a^	Change from Pre-Supplementation ^b^	*p* Value ^c^	Change from Baseline ^b^	*p* Value ^c^
No-metformin-use	47	100.3 ± 31	44	97.7 ± 28.5	44	96.7 ± 30.6	−2.6	0.6654	−1.0	0.2289
Metformin-use										
Overall	72	83.6 ± 21	70	82.5 ± 20.5	71	76.4 ± 22.3	−1.1	0.7701	−6.1	0.0021 **
<1000 mg/day	16	87.3 ± 16.2	16	87.5 ± 17.6	16	86.9 ± 19.8	0.2	0.9219	−0.6	0.8858
≥1000 mg/day	56	82.5 ± 22.2	54	81 ± 21.2	55	73.4 ± 22.2	−1.5	0.7459	−7.6	0.0008 ***

^a^ Data are expressed as mean ± SD for available participants at each specified time point. ^b^ Changes from pre-supplementation (−3 months) and baseline (0 month) represent absolute mean differences (Δ). ^c^ *p*-values were calculated using paired *t*-tests with Bonferroni correction for multiple comparisons to evaluate differences between pre-supplementation (−3 months) and baseline (0 month), as well as baseline (0 month) and post-supplementation (+3 months). Sample sizes at each time point reflect complete paired data available from routine clinical testing. ** *p* < 0.01, *** *p* < 0.001, compared to the baseline.

**Table 6:** Effects of BMP on HDL-C (mg/dL) in participants with or without metformin treatment.

Group	Case No.	Pre-Supplementation ^a^	Case No.	Baseline	Case No.	Post-Supplementation ^a^	Change from Pre-Supplementation ^b^	*p* Value ^c^	Change from Baseline ^b^	*p* Value ^c^
No-metformin-use	47	54.3 ± 14.2	44	54.5 ± 14.9	38	54.1 ± 14.2	0.2	0.0809	−0.4	0.0567
Metformin-use										
Overall	72	45.8 ± 11.7	72	44.2 ± 10.4	67	49.6 ± 50.8	−1.6	0.0569	5.4	0.4298
<1000 mg/day	16	48.4 ± 11.4	16	45.4 ± 8.7	15	45.1 ± 8.1	−3.0	0.0207 *	−0.3	0.3172
≥1000 mg/day	56	45.1 ± 11.8	56	43.8 ± 10.9	52	50.9 ± 57.6	−1.3	0.1960	7.1	0.7564

^a^ Data are expressed as mean ± SD for available participants at each specified time point. ^b^ Changes from pre-supplementation (−3 months) and baseline (0 month) represent absolute mean differences (Δ). ^c^ *p*-values were calculated using paired *t*-tests with Bonferroni correction for multiple comparisons to evaluate differences between pre-supplementation (−3 months) and baseline (0 month), as well as baseline (0 month) and post-supplementation (+3 months). Sample sizes at each time point reflect complete paired data available from routine clinical testing. * *p* < 0.05, compared to the baseline.

## Data Availability

The preclinical animal experiment datasets are available from the corresponding authors upon reasonable request. The individual-level data generated and analyzed in this study are not publicly available due to legal and ethical restrictions. However, aggregated clinical data may be made available by the corresponding author upon request.

## Supplementary Materials

The following supporting information can be downloaded at: https://www.mdpi.com/article/10.3390/nu18162649/s1, Figure S1. Base peak chromatogram (A) and total ion chromatogram (B) of BMP after *in vitro* digestion, analyzed by LC–ESI–MS/MS.

## Abbreviations

The following abbreviations are used in this manuscript:

AGE	Advanced glycation end-products
AMPK	AMP-activated protein kinase
ANOVA	Analysis of variance
BMI	Body mass index
BMP	Bitter melon blood-sugar-modulating protein
CI	Confidence intervals
DEG	Differentially expressed gene
FDR	False discovery rate
FoxO	Forkhead box O
GLUT4	Glucose transporter type 4
HbA1c	Glycated hemoglobin A1c
HDL-C	High-density lipoprotein cholesterol
HIF-1	Hypoxia-inducible factor-1
IR	Insulin receptor
IRB	Institutional Review Board
KEGG	Kyoto Encyclopedia of Genes and Genomes
LC-ESI-MS/MS	Liquid chromatography-electrospray ionization-tandem mass spectrometry
LDL-C	Low-density lipoprotein cholesterol
PI3K	Phosphatidylinositol 3′-kinase
PPAR	Peroxisome proliferator-activated receptor
RAGE	Receptor for advanced glycation end-products
SDS-PAGE	Sodium dodecyl sulfate-polyacrylamide gel electrophoresis
SD	Standard deviation
T1DM	Type 1 diabetes mellitus
T2DM	Type 2 diabetes mellitus
