# Supplementary material for: Glycemic and Lipid Outcomes Associated with Bitter Melon Blood-Sugar-Modulating Protein and Metformin Use: A Translational Preclinical and Retrospective Study
Source: Nutrients. 2026 Aug 13;18(16):2649. doi: 10.3390/nu18162649 (PMC13516629; doi:10.3390/nu18162649)

Supplementary Figure S1. Base peak chromatogram (A) and total ion chromatogram (B) of BMP after *in vitro* digestion, analyzed by LC–ESI–MS/MS.

(A)

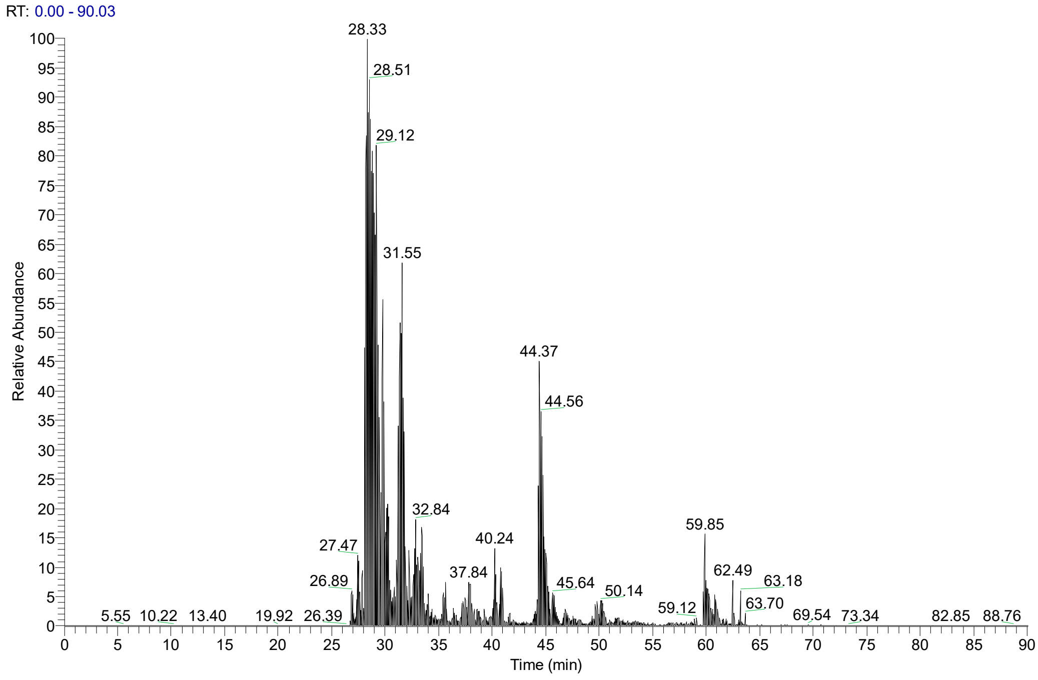

(B)

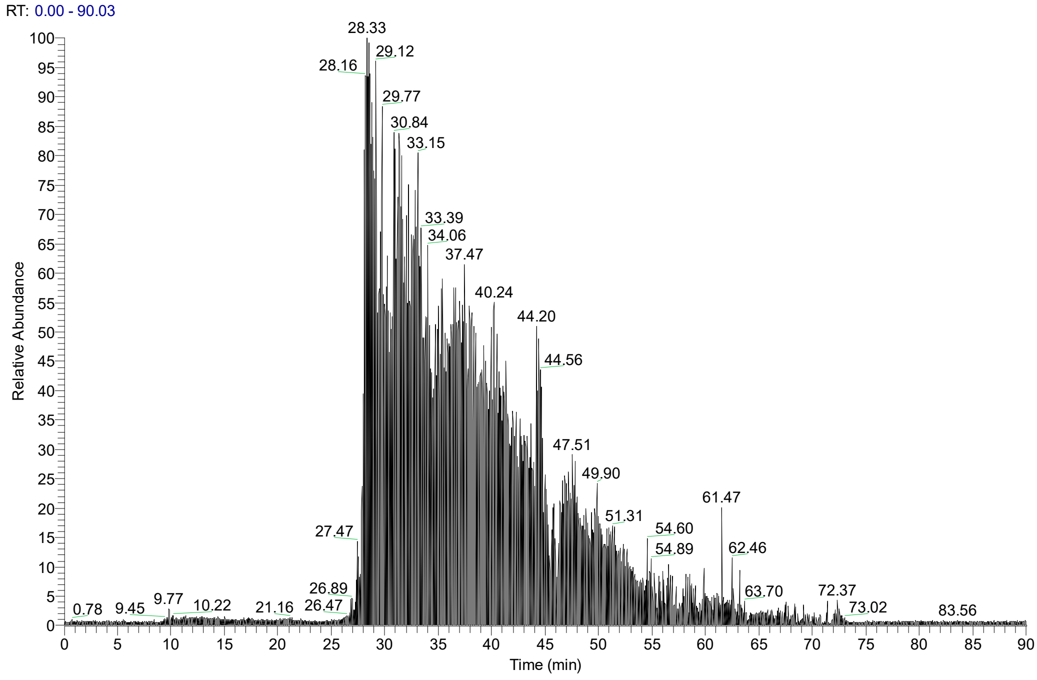

Supplement: Supplementary file 1 [file nutrients-18-02649-s001.zip › nutrients-4455025-supplementary.pdf]
